# Effects of COVID-19 vaccination on clinical outcomes in patients hospitalized in Internal Medicine during Omicron variant spreading.

**DOI:** 10.1007/s11739-022-03185-5

**Published:** 2023-01-13

**Authors:** Luca Colangeli, Gianna Aprile, Clara Carcone, Monica D’Adamo, Emanuela Medda, Paolo Sbraccia, Valeria Guglielmi

**Affiliations:** 1 Department of Systems Medicine, University of Rome Tor Vergata, Via Montpellier 1, 00133 Rome, Italy; 2 Internal Medicine Unit and Obesity Center, University Hospital Policlinico Tor Vergata, Rome, Italy; 3 Centre for Behavioural Sciences and Mental Health, Istituto Superiore Sanità, Rome, Italy

**Keywords:** Internal Medicine, COVID-19, SARS-CoV-2, Omicron, Variant, Vaccination

Dear Editor,

All over the world, mass vaccination is playing a fundamental role in contrasting the novel coronavirus disease 2019 (COVID-19) pandemic. Clinical trials have demonstrated that vaccines are safe and effective in preventing severe disease and death; [1–4] however the emergence of new viral variants such as beta (B.1.351) and delta (B.1.617.2) raised the issues of enhanced transmissibility and immune escape, determining numerous cases of re-infections. [5] In December 2021, delta variant was the predominant one, progressively substituted by omicron (B.1.1.529 [BA.1]) and omicron sub-variants (BA.2, BA.2.12.1, BA.4, and BA.5) at the beginning of 2022. [6] To date, these are the most antigenically different variants compared to the original strain of SARS-CoV-2, presenting at least 32 mutations in spike protein, [7] which determine an important capacity to escape antibodies and a reduction in vaccine effectiveness. [8]

Internal Medicine Units (IMUs), that usually host older patients with multiple chronic diseases, had a significant role in the struggle against COVID-19. Corradini et al. reported the results of a nationwide registry during the so-called first wave of the pandemic, finding in age and number of pre-existing comorbidities the main factors associated with in-hospital death. [9] In our previous study, we have found that not only age and malignancies, but underweight and inflammation levels as well were predictors of worse outcomes (death or mechanical ventilation). [10]

However, little is known about the clinical characteristics and the outcomes of patients hospitalized in Internal Medicine in this new context of mass vaccination and predominance of new viral variants.

The main aim of this study was to evaluate the association of vaccination status and clinical outcomes (interstitial pneumonia, death, and positivity duration) in patients admitted to IMU with SARS-CoV-2 infection when delta and omicron variants were spreading, during the so-called fourth wave of COVID-19 pandemic in Italy.

In this monocentric, retrospective, observational study, all patients admitted to our IMU, at the University Hospital Policlinico Tor Vergata (Rome, Italy), between December 28th, 2021, and April 30th, 2022 were enrolled. All included patients had SARS-CoV-2 infection diagnosis based on real-time reverse transcriptase-polymerase chain reaction (RT-PCR) test on nasopharyngeal swab. Demographic characteristics (age and sex), comorbidities, vaccination status, clinical data and outcomes were collected for all patients from their admission to Emergency Department (ED) up to their discharge at home, transfer to other no-COVID Units or death. Positivity duration was defined as the number of days between the first nasopharyngeal swab tested positive for SARS-CoV-2 and the first negative nasopharyngeal swab. This observational study was based on medical records, patient confidentiality was protected by assigning an anonymous identification code and electronic data were stored in a password-protected computer.

Chi-square test and Mann–Whitney test were applied as appropriate to assess differences between groups. Multivariate logistic regression was used to evaluate the association between different considered vaccination status and outcomes (interstitial pneumonia, mortality and positivity duration ≥ 21 days) considering the effect of age and gender. Adjusted odds ratio (OR) and related 95% confidence intervals (95% CI) were estimated and OR was considered significant when 1.0 was not included in 95% CI. Statistical analyses were conducted using STATA software (version 16.0; StataCorp, College Station, TX, USA).

The study protocol was approved by the ethical committee of Policlinico Tor Vergata.

The study includes 84 patients (41 males and 43 females; mean age 76.1 ± 14.3 years). Baseline characteristics of patients grouped by the main outcomes evaluated (interstitial pneumonia and death) are presented in Table 1.

**Table 1:** Baseline characteristics of patients and vaccination status grouped for outcomes evaluated.

	No IP	IP	*p *value	No death	Death	*p* value
	(*n* = 40)	(*n* = 43)		(*n* = 65)	(*n* = 19)	
	Mean ± SD or *N* (%)	Mean ± SD Or *N* (%)		Mean ± SD or *N* (%)	Mean ± SD or *N* (%)	
Baseline characteristics						
Age (years)	73.0 ± 15.7	79.0 ± 12.5	0.06	73.0 ± 14.6	86.0 ± 7.9	< 0.001
Males	18 (45.0%)	22 (51.2%)	ns	32 (49.2%)	9 (47.4%)	ns
SARS-CoV-2 symptoms	13 (32.5%)	17 (39.5%)	ns	25 (38.5%)	5 (26.3%)	ns
Number of comorbidities	4.2 ± 2.1	4.8 ± 2.0	ns	4.6 ± 2.1	4.4 ± 2.2	ns
Vaccination status						
At least one dose	37 (92.5%)	33 (76.7%)	< 0.05	56 (86.2%)	15 (78.9%)	ns
Booster dose	22 (55.0%)	22 (51.2%)	ns	36 (55.4%)	9 (47.4%)	ns

*IP* interstitial pneumonia, *SD* standard deviation.

One patient did not perform CT scan and data on interstitial pneumonia are therefore missing.

At the Emergency Department (ED) arrival, 35.7% of patients (*n* = 30) reported symptoms related to SARS-CoV-2 infection. Dyspnoea was the most frequent symptom (23.8%), followed by asthenia (15.5%) and fever (14.3%). Most of the patients (*n* = 54, 64.3%) accessed to ED for symptoms not related to SARS-CoV-2 infection, that was detected at a routine nasopharyngeal swab.

Almost all patients (98.8%) reported at least one comorbidity, with a mean of 4.5 ± 2.1 comorbidities for each patient. The main comorbidities were hypertension (75.0%), dyslipidaemia (54.8%), heart failure (42.9%), central nervous system disorders (36.9%), atrial fibrillation (31%), respiratory diseases (27.4%), coronary artery disease (26.2%), type 2 diabetes (25%), peripheral artery disease (19%), chronic kidney disease (17.9%), malignant neoplasms (17.9%), previous stroke (16.7%), obesity (13.1%), haematologic diseases (9.5%), immunosuppression (8.3%), rheumatologic diseases (8.3%) and chronic liver disease (3.5%).

Regarding vaccination status, 84.5% of patients (*n* = 71) received at least one dose of one of the approved COVID-19 vaccines available in Italy and 53.7% (*n* = 45) received booster dose. Interestingly, only 3 patients (3.6%) reported previous SARS-CoV-2 infection (more than 120 days before hospital admission) and just 1 of them received COVID-19 vaccination after the primary infection.

Altogether, 19 patients (22.6%) died during IMU hospital stay, 16 patients (19.0%) were transferred to other no-COVID Units after resolution of SARS-CoV-2 infection (defined as two consecutive negative nasopharyngeal swab), 49 patients (58.4%) were discharged at home.

Age was significantly associated with mortality (OR 1.10, 95% CI 1.04 – 1.19; *p* = 0.002) and patients older than 75 years showed a 7-fold mortality risk compared to younger patients. On the contrary, we did not find significant difference in mortality based on number of comorbidities.

Of the 19 patients who died, 78.9% received at least one dose of vaccine (9 of them received booster dose), while 21.1% were unvaccinated. Prevalence of mortality by vaccination status is illustrated in Figure 1. At multivariate logistic regression analysis, neither vaccination nor booster dose were associated to lower mortality in our cohort. However, although the estimates did not reach statistical significance, the effect size points toward a reduction in the risk and the lack of statistical significance may be due to the small sample size of the study. Indeed, the mortality risk was shown to be halved in vaccinated subjects (OR 0.52, 95% CI 0.11 – 2.40, *p* = 0.40; and OR: 0.35, 95% CI 0.06 – 2.01, *p* = 0.24, for vaccination and booster, respectively).

**Fig. 1:**
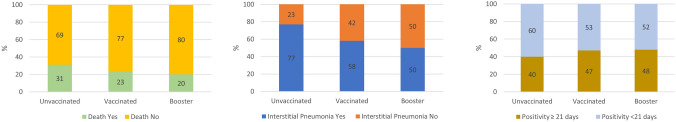
Prevalence of mortality, interstitial pneumonia and positivity duration in the study population by vaccination status.

After the diagnosis of SARS-CoV-2 infection, all patients (except one, *n* = 83) performed chest CT scan without contrast, which revealed interstitial pneumonia in 51.8% of cases (*n* = 43). Receiving at least one dose of vaccine had a protective effect against the occurrence of interstitial pneumonia (OR 0.23, CI 95% 0.05 – 0.94; *p *= 0.04), and a similar, although not significant, trend for booster dose (OR 0.24, CI 95% 0.06 – 1.11; *p *= 0.07) was observed.

Positivity duration was evaluated in 42 patients (as we did not include in this analysis patients who died and patients who were discharged at home with a still positive nasopharyngeal swab) and mean positivity duration was 22.1 ± 12.6 days. Prevalence of positivity duration ≥ 21 days by vaccination status is illustrated in Figure 1. Before and after adjustment for age and sex, vaccination and booster dose were not significantly associated to positivity duration longer than 21 days (OR 0.44, 95% CI 0.04 – 4.86; *p* = 0.51; and OR 0.57, 95% CI 0.05 – 6.89; *p* = 0.66, respectively).

What we can observe is that only 37.5% of patients referred to ED for COVID-19 related symptoms, reflecting the fact that infection diagnosis is more frequently incidental compared to previous pandemic waves, [9] and that those who received at least one dose of vaccine had a significantly lower incidence of interstitial pneumonia. Therefore, mass vaccination is changing clinical presentation of in-hospital patients with SARS-CoV-2 infection. Our results on mortality must be interpreted in the context of IMU, where patients are older, with a relevant number of comorbidities, hospitalized for potentially life-threatening acute diseases but without a severe or critical COVID-19 clinical presentation. In fact, our mortality rate is in line with what was recently reported by Lenti et al (27%). [11] Moreover, all patients were treated with prophylactic dose of low molecular weight heparin, dexamethasone, monoclonal antibodies (casirivimab/imdevimab or sotrovimab) and antiviral therapy (remdesivir) when clinically indicated, in accordance with the best available scientific evidence. [12] Treatment with antiviral drugs and monoclonal antibodies [13, 14] may have had a relevant impact on mortality, not evaluated in this study. Finally, a reduction in risk of death *per se* after omicron infection as compared with previous variants might be also considered, [15] independently from vaccination status. For what concerns positivity duration, we did not observe differences between vaccinated and unvaccinated patients and we registered a longer positivity duration than previously reported with Omicron variant in younger people. [16] These observations may suggest that our population had a lower capacity of reducing viral load, potentially impaired by age and comorbidities.

This study has some limitations. First of all, patients admitted in our IMU presented a mild-to-moderate COVID-19 clinical presentation, as patients more severely or critically ill were rather admitted to Pneumology or Intensive Care Units, and this may represent a selection bias. Secondly, even if our study was conducted when spreading of delta and omicron variants was epidemiologically proved, we did not perform viral genome sequencing. Finally, this was a monocentric, retrospective study with a limited sample size and its results cannot be extended to general population.

In conclusion, SARS-CoV-2 clinical presentation is changing. In the context of Internal Medicine units, receiving at least one dose of vaccine resulted to be protective against the occurrence of interstitial pneumonia. In our study, vaccination status was not significantly associated to lower mortality neither to reduction of positivity duration, but the small sample size is insufficient to draw firm conclusions. The emergence of new viral variants makes it necessary further and continuously updated real-world studies to evaluate COVID-19 pandemic evolution and how to face it.

## Data Availability

The data presented in this study are available on request to the corresponding author.

## References

[1] Polack FP (2020). Safety and efficacy of the BNT162b2 mRNA Covid-19 vaccine. N Engl J Med.

[2] Baden LR (2021). Efficacy and safety of the mRNA-1273 SARS-CoV-2 vaccine. N Engl J Med.

[3] Sadoff J (2021). Safety and efficacy of single-dose ad26cov2s vaccine against covid-19. N Engl J Med.

[4] Dunkle LM (2022). Efficacy and safety of NVX-CoV2373 in adults in the United States and Mexico. N Engl J Med.

[5] Mohsin M, Mahmud S (2022). Omicron SARS-CoV-2 variant of concern: a review on its transmissibility, immune evasion, reinfection, and severity. Medicine.

[6] Barouch DH (2022). Covid-19 vaccines - immunity, variants Boosters. N Engl J Med.

[7] Tian D (2022). The emergence and epidemic characteristics of the highly mutated SARS-CoV-2 Omicron variant. J Med Virol.

[8] Tseng HF (2022). Effectiveness of mRNA-1273 against SARS-CoV-2 Omicron and Delta variants. Nat Med.

[9] Corradini E (2021). Clinical factors associated with death in 3044 COVID-19 patients managed in internal medicine wards in Italy: results from the SIMI-COVID-19 study of the Italian Society of Internal Medicine (SIMI). Intern Emerg Med.

[10] Guglielmi V (2022). Inflammation, underweight, malignancy and a marked catabolic state as predictors for worse outcomes in COVID-19 patients with moderate-to-severe disease admitted to internal medicine unit. PLoS One.

[11] Lenti MV (2022). Determinants of COVID-19-related mortality in an internal medicine setting. Intern Emerg Med.

[12] AIFA. *Trattamenti utilizzabili nei pazienti COVID-19 nel setting ospedaliero.* 2022 26/NOV/2022]; Available from: https://www.aifa.gov.it/documents/20142/1269602/SOC_ospedaliera_03_06.06.2022.pdf.

[13] Razonable RR (2022). Real-world clinical outcomes of bebtelovimab and sotrovimab treatment of high-risk persons with coronavirus disease 2019 during the omicron epoch. Open Forum Infect Dis.

[14] Ghazanfar H (2022). Outcomes of monoclonal antibody infusion treatment during delta (B16172) and omicron (B11529) COVID 19 variant surges among vaccinated and unvaccinated patients. Health Serv Insights.

[15] Ward IL (2022). Risk of covid-19 related deaths for SARS-CoV-2 omicron (B1.1.529) compared with delta (B.1.617.2): retrospective cohort study. Bmj..

[16] Kojima N, Roshani A, Klausner JD (2022). Duration of COVID-19 PCR positivity for Omicron vs earlier variants. J Clin Virol Plus.

